# Standard laboratory tests to identify older adults at increased risk of death

**DOI:** 10.1186/s12916-014-0171-9

**Published:** 2014-10-07

**Authors:** Susan E Howlett, Michael RH Rockwood, Arnold Mitnitski, Kenneth Rockwood

**Affiliations:** Department of Medicine, Dalhousie University, Halifax, Nova Scotia Canada; Departments of Pharmacology and Medicine (Division of Geriatric Medicine), Dalhousie University, 5850 College Street, Sir Charles Tupper Medical Building, Halifax, Nova Scotia B3H 4R2 Canada; Department of Physiology, Institute of Cardiovascular Sciences, University of Manchester, Oxford Road, Manchester, M13 9PL UK; Department of Medicine (Divisions of Geriatric Medicine and Neurology), Dalhousie University, Halifax, Nova Scotia Canada; Department of Geriatric Medicine and Institute of Brain, Behaviour and Neurosciences, University of Manchester, Oxford Road, Manchester, M13 9PL UK

**Keywords:** Frailty, Frailty index, Deficit index, Deficit accumulation, Biomarkers, Aging

## Abstract

**Background:**

Older adults are at an increased risk of death, but not all people of the same age have the same risk. Many methods identify frail people (that is, those at increased risk) but these often require time-consuming interactions with health care providers. We evaluated whether standard laboratory tests on their own, or added to a clinical frailty index (FI), could improve identification of older adults at increased risk of death.

**Methods:**

This is a secondary analysis of a prospective cohort study, where community dwelling and institutionalized participants in the Canadian Study of Health and Aging who also volunteered for blood collection (n = 1,013) were followed for up to six years. A standard FI (FI-CSHA) was constructed from data obtained during the clinical evaluation and a second, novel FI was constructed from laboratory data plus systolic and diastolic blood pressure measurements (FI-LAB). A combined FI included all items from each index. Predictive validity was tested using Cox proportional hazards analysis and discriminative ability by the area under receiver operating characteristic (ROC) curves.

**Results:**

Of 1,013 participants, 51.3% had died by six years. The mean baseline value of the FI-LAB was 0.27 (standard deviation 0.11; range 0.05 to 0.63), the FI-CSHA was 0.25 (0.11; 0.02 to 0.72), and the combined FI was 0.26 (0.09; 0.06 to 0.59). In an age- and sex-adjusted model, with each increment in the FI-LAB, the hazard ratios increased by 2.8% (95% confidence interval 1.02 to 1.04). The hazard ratios for the FI-CSHA and the combined FI were 1.02 (1.01 to 1.03) and 1.04 (1.03 to 1.05), respectively. The FI-LAB and FI-CSHA remained independently associated with death in the face of the other. The areas under the ROC curves were 0.72 for FI-LAB, 0.73 for FI-CSHA and 0.74 for the combined FI.

**Conclusions:**

An FI based on routine laboratory data can identify older adults at increased risk of death. Additional evaluation of this approach in clinical settings is warranted.

**Electronic supplementary material:**

The online version of this article (doi:10.1186/s12916-014-0171-9) contains supplementary material, which is available to authorized users.

## Background

Frailty is an important problem for aging societies [1]. Increasingly, there is a sense that societies need to begin frailty screening and assessment [2], even though lack of consensus about just how to do this is acknowledged [3]. Frail older adults who become acutely ill are seen as being at particular risk, especially if exposed to the hazards of routine hospital care [4–6] without mitigation by specialized geriatric interventions [7].

Reflecting the lack of consensus, various scales to measure frailty are used [8]. One common approach is to quantify frailty with a frailty index (FI), based on the accumulation of health deficits [9–11]. These deficits can be symptoms, clinical signs, diseases, laboratory abnormalities or other measures [11]. An FI score is achieved by counting the number of deficits in an individual and dividing by the total number of deficits measured to produce a score between 0 and 1; a higher score indicates greater frailty [10,11]. For a deficit to be included in an FI it must be shown that its prevalence increases with age, that it does not become too prevalent at some younger age and that it is associated with adverse outcomes [11]. Thus, an FI score provides a quantitative measure of health status and characterizes the risk of adverse outcomes, including death.

In clinical care, the ideal frailty screening tool would quantify frailty based on data that are collected routinely. In the hospital setting for example, admission typically is associated with a large number of blood tests and routine physical measures (for example, blood pressure) that require minimal participation by patients. Recent work on measuring frailty in mice suggests that, in combination, many such tests show minor abnormalities that, in the aggregate contribute to risk [12]. We wondered whether this might also obtain with routine physical assessment and laboratory test data that are often collected clinically. For this reason, in a re-analysis of data from the clinical examination conducted during the first wave of the Canadian Study of Health and Aging (CSHA), our objectives were: 1) to develop an FI based only on routine physical and laboratory tests (FI-LAB); 2) to validate the FI-LAB in relation to age, sex and distribution; and 3) to test its predictive value in relation to death. Here, we show that an FI based on routine laboratory tests identifies older adults at an increased risk of death.

## Methods

### Participants, setting and sample

The CSHA is a cohort study of health problems of older adults (aged 65+ at baseline). Community-dwelling participants were screened using a cognitive test (the Modified Mini-Mental State examination-3MS) [13]. As detailed elsewhere [14], those who screened positive (n = 1,614) and a comparison group included in a separate risk factor study (n = 731) were invited to a clinical examination; 1,659 completed that examination. Institutionalized participants were not screened but went straight to a clinical examination (n = 1,255); mortality data were obtained at the five year follow-up in 1996/1997 [15]. The clinical examination included a history from participants and/or knowledgeable informants, as well as hospital records and routine clinical laboratory data, where available. Of the 2,914 participants who had a clinical examination, the present study used a subset from both community-dwelling (n = 683) and institutionalized (n = 330) participants, for whom there were sufficient items to construct an FI relevant to both samples and who in addition had laboratory data. These 1,013 subjects represented 74% of the 1,375 clinical interview participants who had laboratory data.

### Health measures/deficits

First, a standard FI (FI-CSHA) was constructed from data obtained during the clinical evaluation as described in detail in previous studies by our group (for example, [11,16,17]). The FI was composed of up to 38 variables used in the initial CSHA clinical examination [see Additional file 1: Table S1]. Each self-reported medical condition, disease history, symptom, and health rating variable satisfied the criteria for being a deficit as described previously [11]. An FI score was calculated where more than 60% of the variables were available for a given individual. Although clinical data were available for 1,375 individuals, 362 were excluded from analysis due to missing data to yield a total sample size of 1,013.

Next, we developed an FI (the FI-LAB) of up to 23 variables based on 21 routine blood tests plus measured systolic and diastolic blood pressure (Table 1). This latter, novel FI was called the ‘laboratory FI’ or ‘FI-LAB’. The FI-LAB was constructed by first coding each variable as either 0 or 1, where ‘0’ indicates that values are within the normal cut-offs and ‘1’ indicates that values are either above or below the normal cut off values illustrated in Table 1. An FI-LAB score was calculated only if more than 70% of the lab variables were available for a given individual. Each person’s FI-LAB score was calculated as the number of deficits present divided by the total number of deficits measured. For example, an individual with no deficits would have an FI-LAB score of 0, whereas one in whom all possible deficits were present would have the theoretical maximal FI-LAB score of 1. In a separate analysis, we added the deficit scores in the FI-LAB and the deficit scores in the FI-CSHA and divided by the new total to produce a ‘combined’ FI score.

**Table 1:** **Clinical and laboratory data used to construct the FI-LAB**

**Variable** ^**a**^	**Low cut-off**	**High cut-off**
**Albumin (g/L)**	32	45
**AST (SGOT; IU/L)**	8	33
**BP, supine systolic (mmHg)**	90	140
**BP, supine diastolic (mmHg)**	60	90
**Calcium (mM)**	2.3	2.7
**Creatinine (μM)**	53	106
**Folate (nM)**	11	57
**Folate, RBC (nM)**	376	1450
**Glucose, fasting (mM)**	3.9	6.1
**Hemoglobin (g/L)** ^**b**^	135	180
**Mean corpuscular volume (fL)**	80	96
**Phosphatase, alkaline (IU/L)**	20	130
**Phosphorus, inorganic (mM)**	0.74	1.52
**Potassium (mM)**	3.8	5
**Protein, total (g/L)**	60	78
**Sodium (mM)**	136	142
**TSH (μIU/L)**	0.5	5
**Thyroxine (T4; nM)**	71	161
**T4, Free (pM)**	12	30
**Urea (mM)**	2.9	8.2
**VDRL**	0	0
**Vitamin B12 (pg/L)**	118	701
**White blood cells (number/L)**	1.8 × 10^9^	7.8 × 10^9^

^a^Normal reference values for blood work were from Henry [18]. Reference values for normal blood pressure were from Jones *et al*. [19] and Pickering *et al*. [20]. ^b^Note that normal references values for hemoglobin differed between the sexes so for women, the low cut-off was 120 g/L and the high cut-off was 160 g/L. AST, aspartate aminotransferase; BP, blood pressure; FI-LAB, Laboratory frailty indes; RBC, red blood cells; TSH, thyroid-stimulating hormone; VDRL, Venereal Disease Research Laboratory.

### Outcomes

The major outcome was survival (that is, died or survived) over up to six years of follow-up. Decedent data were obtained from the Registrar of Vital Statistics in each province as well as from interviews with spouses or next of kin of study participants who had died.

### Standard protocol approvals, registrations and patient consents

Data collection was approved by the ethics review process for the CSHA. Approval for the secondary analyses came from the Research Ethics Committee of the Capital District Health Authority, Halifax, Nova Scotia, Canada. All participants (or designates) signed informed consent forms.

### Statistical analysis

Demographic and clinical characteristics were expressed as either a percentage of the total sample or as the mean ± SD, or in some cases, as the mean ± SE. Density distributions for each of the FI-CSHA, FI-LAB and combined FI scores were plotted and the median, minimal and maximal values were calculated. The age-specific distribution of each FI was estimated by plotting the mean of the natural logarithm of the FI score at each year of age from age 65 onwards; data were fitted with a linear regression function, and the fit, slope and intercept were evaluated. The relationship between the FI-CSHA and the FI-LAB was investigated by first calculating the mean of the FI-CSHA in increments of 0.05. Then the average FI-LAB values were plotted as a function of the FI-CSHA for each increment and the resulting line was fitted by linear regression. The distribution of the FI by months to death was evaluated with Kaplan-Meier survival analysis. For purposes of presentation, the Kaplan-Meier survival curves were presented for four grades for each FI (<0.10, 0.10 to 0.22, 0.23 to 0.45 and >0.45). To investigate the impact of FIs on mortality, Cox proportional hazard regression models adjusted for age and sex were used. The FI values were converted to integers between 0 and 100 by rounding them after multiplying them by 100, giving equal percent increments.

Some analyses were performed using codes developed in Matlab (version 2007, Mathworks Inc.). Additional analyses were performed either with SPSS (IBM SPSS Statistics, Version 21) or Sigma Plot 11.0 (Systat Software, Inc., Point Richmond, CA, USA). Graphs were created with Sigma Plot 11.0. The statistical significance level was set at *P* <0.05.

## Results

Of the 1,375 people with both clinical examinations and laboratory data, complete data were available on 1,013, of whom vital status was known for 986 (97.3%; Additional file 1: Figure S1). Selected demographic and clinical characteristics of the study population, subdivided by grades of frailty for both the FI-CSHA and the FI-LAB, are illustrated in Table 2. The mean frailty scores increased with age for both frailty measures. Mean FI-CSHA scores increased from 0.07 ± 0.02 in the least frail group to 0.50 ± 0.05 in the frailest group (Table 2). Similar results were seen when the FI-LAB scores were used to stratify frailty. The average FI-LAB values increased from 0.08 ± 0.02 in the group with the lowest scores to 0.50 ± 0.04 in the group with the highest frailty (Table 2). Of note, the proportion of women with low FI scores was much higher when frailty was stratified by FI-LAB scores compared to the FI-CSHA. The mean combined FI scores also increased from 0.08 ± 0.01 in the group with the lowest scores to 0.50 ± 0.04 in the group with the highest frailty scores (Table 2). The characteristics of the 372 excluded cases (mean (±SD) FI-CSHA = 0.26 ± 0.12; mean age = 81.9 ± 7.9 years; 64.4% women) were similar to those of the 1,013 included cases.

**Table 2:** **Baseline demographic and clinical characteristics and mortality by grades of frailty**

**Baseline Characteristic**	**Grades of the FI**			
**FI-CSHA**	**<0.10**	**0.10 to 0.22**	**0.23 to 0.45**	**>0.45**
	**Number = 78**	**Number = 349**	**Number = 539**	**Number = 47**
Mean age, years (±SD)	76.2 ± 6.1	80.2 ± 7.2	82.1 ± 7.1	83.3 ± 5.6
Mean FI-CSHA (±SD)	0.073 ± 0.019	0.164 ± 0.034	0.309 ± 0.062	0.500 ± 0.051
Women (%)	48.7	59.7	65.1	53.2
Institutionalized (%)	1.3	25.5	45.7	4.3
Mortality (%)	25.6	39.8	60.1	70.2
**FI-LAB**	**<0.10**	**0.10 to 0.22**	**0.23 to 0.45**	**>0.45**
	**Number = 56**	**Number = 255**	**Number = 645**	**Number = 57**
Mean age, years (±SD)	77.8 ± 6.6	80.1 ± 7.3	81.5 ± 7.1	83.6 ± 6.8
Mean FI-LAB (±SD)	0.083 ± 0.019	0.168 ± 0.027	0.310 ± 0.063	0.503 ± 0.040
Women (%)	60.7	67.8	60.5	47.4
Institutionalized (%)	19.6	30.2	35.0	43.9
Mortality (%)	19.6	44.7	54.4	77.2
**Combined FI**	**<0.10**	**0.10 to 0.22**	**0.23 to 0.45**	**>0.45**
	**Number = 15**	**Number = 346**	**Number = 635**	**Number = 17**
Mean age, years (±SD)	74.5 ± 4.7	79.0 ± 7.0	82.3 ± 7.0	82.6 ± 6.0
Mean combined FI (±SD)	0.080 ± 0.013	0.173 ± 0.033	0.303 ± 0.055	0.501 ± 0.039
Women (%)	53.3	58.1	64.3	41.2
Institutionalized (%)	0	19.9	42.4	5.9
Mortality (%)	6.7	33.5	61.1	88.2

FI, frailty index; FI-CSHA, standard frailty index; FI-LAB, laboratory frailty index; SD, standard deviation.

**Figure 1:**
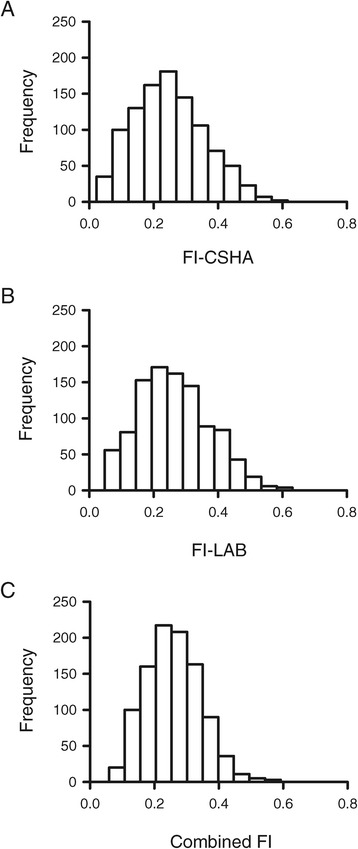
**Frequency distributions for the FI-CSHA and the FI-LAB. A)** The frequency distribution for the FI-CSHA data was somewhat skewed to the left, with a median of 0.24 and a long right tail. The maximum FI-CSHA score was 0.72. **B)** Histogram showing the frequency distribution for the FI-LAB data collected in this study. The distribution had a median FI-LAB value of 0.27 and the maximum observed FI-LAB score was 0.63. **C)** The distribution of the combined FI scores was slightly skewed to the left, with a median value of 0.26 and a maximum of 0.59. The value of n = 1,013 participants in each group. FI-CSHA, standard frailty index; FI-LAB, laboratory frailty index.

To compare the distribution of FI scores for the three different FI instruments used in this study, frequency distributions for each were plotted. Figure 1A shows a frequency distribution of the FI-CSHA scores obtained for the cohort investigated in this study. The distribution was slightly skewed to the left, with a mean of 0.25 ± 0.11 (±SD) and a median of 0.24. The minimum FI-CSHA score observed was 0.02 while the maximum was 0.72 (Figure 1A), consistent with the idea that there is a sub-maximal limit to frailty near 0.7. The frequency distribution for the FI-LAB scores is shown in Figure 1B. This distribution had a mean of 0.27 ± 0.12 (±SD) and a median of 0.27. The minimal and maximal FI-LAB scores were 0.05 and 0.63, respectively (Figure 1B). Figure 1C shows that the frequency distribution for the combined FI scores was similar to the distribution of the two parent index scores. This distribution was slightly skewed to the left with a mean of 0.26 ± 0.09 (±SD), a median of 0.25 and minimal and maximal scores of 0.06 and 0.59, respectively (Figure 1C).

**Figure 2:**
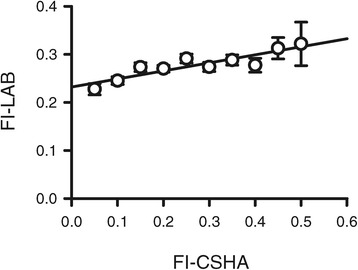
**Relationship between the FI-CSHA and the FI-LAB.** FI-LAB values were plotted as a function of the FI-CSHA scores. The FI-CSHA scores were pooled and the means (±SEM) are shown in increments of 0.05. The FI-LAB increased as FI-CSHA scores increased (*P* <0.001). The data were fit with a linear regression as described in the methods and were a good fit to a straight line (r^2^ = 0.81). FI-CSHA, standard frailty index; FI-LAB, laboratory frailty index; SEM, standard error of the mean.

The log of each FI score increased linearly with age (data not shown). The r^2^ values were 0.57 for the FI-CSHA, 0.62 for the FI-LAB and 0.69 for the combined FI. Regression lines fitted through the data had slopes of 0.015, 0.012 and 0.013 for the FI-CSHA, the FI-LAB and the combined FI, respectively. To determine whether the FI-CSHA scores and the FI-LAB scores were linearly related, the FI-CSHA scores were plotted as a function of the FI-LAB scores (Figure 2). The average FI-LAB scores increased as the FI-CSHA increased; this relationship was a very good fit to a straight line (r^2^ = 0.81).

**Figure 3:**
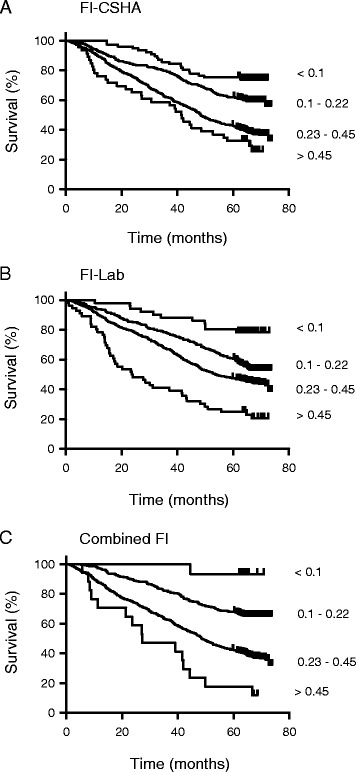
**Kaplan-Meier survival curves for grades of the FI. A)** Survival over the course of the study plotted as a function of grades of the FI-CSHA. The least frail group (frailty score <0.10) showed little mortality over the course of the study whereas the most frail group (frailty score >0.45) showed very high mortality. Differences between groups were statistically significant between all four grades of frailty when analyzed with a log-rank test (*P* <0.05). **B)** Survival curves for grades of frailty assessed by the FI-LAB scores. There were significant differences in survival between subjects at all four levels when FI-LAB scores were used to grade frailty (*P* <0.05; log rank test). **C)** Kaplan-Meier survival curves for ‘combined’ FI scores obtained by merging the FI-CSHA and the FI-LAB scores. Differences in mortality between the four grades of frailty were most evident when the combination FI scores were used (*P* <0.05; log rank test). FI-CSHA, standard frailty index; FI-LAB, laboratory frailty index.

Mortality rates generally increased as the frailty scores rose, although this effect was more marked with the FI-LAB than with the FI-CSHA scores (Table 2). Mortality also increased significantly as the frailty scores increased (Table 2, Figure 3). Note that in an age and sex adjusted model, each contributed independently: the odds ratio for the FI-LAB =1.03, 95% CI =1.01, 1.04, versus FI-CSHA = 1.04, 95%CI = 1.02, 1.05. The combined FI showed the clearest separation of groups by grades of frailty (Figure 3), and was associated with the highest hazard rates in age and sex adjusted models (Table 3). The impact on the discriminative ability of combining both FIs was modest: the area under the receiver operating characteristic (ROC) curve was 0.71 for the FI-CSHA, 0.72 for the FI-LAB and 0.74 for the combined FI (Additional file 1: Figure S2). Nonetheless, together these data show that all three FI scores identified older adults at increased risk of death.

**Table 3:** **Cox proportional hazards regression models for death**

**Variable**	**Beta**	**Standard error**	**Z score**	**Hazard ratio**	**95%** **confidence interval**
**Sex**	−0.404	0.102	−3.989	0.67	0.54 to 0.82
**Age**	0.069	0.008	9.207	1.07	1.06 to 1.09
**FI-CSHA**	0.021	0.004	4.865	1.02	1.01 to 1.03
**Sex**	−0.306	0.102	−3.002	0.74	0.60 to 0.90
**Age**	0.066	0.008	8.753	1.07	1.05 to 1.08
**FI -LAB**	0.028	0.005	5.991	1.03	1.02 to 1.04
**Sex**	−0.355	0.101	−3.505	0.70	0.57 to 0.86
**Age**	0.064	0.008	8.442	1.07	1.05 to 1.08
**Combined FI**	0.040	0.006	7.058	1.04	1.03 to 1.05

FI, frailty index; FI-CSHA, standard frailty index; FI-LAB, laboratory frailty index.

## Discussion

We investigated the properties of an FI made up of information from widely used laboratory tests. That FI (the FI-LAB) had properties similar to other FIs, including the FI-CSHA. The latter consists of up to 38 items from the CSHA clinical examination and corresponds to most of the items used in a Comprehensive Geriatric Assessment, which can be summarized as an FI [5,6,16,17]. Even so, the people classified as at risk by each method differed in interesting ways. Although the distributions of the two FIs were similar, as were their mean ages, fewer people had the lowest FI scores in both FIs. For example, only 15 people had a combined FI score less than 0.10, compared with 78 for the FI-CSHA and 56 for the FI-LAB (Table 2). Of these 15, only 1 died (Figure 3), the lowest mortality rate of the three FIs (combined, FI-LAB and FI-CSHA). The analogous case holds for people with the highest scores in each of the FI-LAB (n = 57) and FI-CSHA (n = 39). Only 17 individuals had the highest scores in both and of these 15 died (Table 2). This 88% five-year mortality was the highest for any FI category. These large differences in mortality in relation to the combined FI categories were observed even though the increase in the area under the curves (AUCs) was modest, suggesting that the middle classifications could be more finely graded. Even so, we have resisted finer grades of the combined FI, on the grounds that, from a clinical point of view, knowing the highest and the lowest risks is most important: people of intermediate risk represent variations on the usual case, and typically receive usual management without useful precision in prognosis. In any case, testing how much information comes from the nature of the added items, and how much comes from their number is best addressed in a different datatset.

Additional questions remain, however, such as whether the smaller number of people with the fewest things, or most things, wrong reflects a specific increase in information from laboratory data, or whether that might be shown simply by increasing the number of items considered in an FI. Here, the distribution of the items in each FI suggests some comparability (and is reassuring in relation to combining items), but their independent contribution in a multivariable model suggests that they are offering independent information. The latter suggests that subclinical information, or at least information more precisely detected with laboratory tests, could offer additional insights. As detailed elsewhere [21,22], frailty that is macroscopically detectable represents the build-up of subcellular, tissue and organ deficits, being damage at those levels that has gone either unremoved or unrepaired. The lethality of any clinical/macroscopic deficits on a background of subclinical/microscopic deficits is suggested by the combined FI hazard rate being the highest, and by the notably diminished survival of the frailest group (FI >0.45) in the combined FI (Figure 3, Panel C, compare with Panels A and B).

Our data must be interpreted with caution. Our sample, although population-based, is not representative. Given that it was drawn from the clinical examination database, it is older and contains proportionally more institutionalized people than the population from which it was drawn. Even within the clinical sample, developing an FI with items that were relevant for both community-dwelling and institutionalized people was a challenge: for example, no one in institutional care is independent in any instrumental activities of daily living (ADLs), and few community-dwelling people have fecal incontinence or behavioral problems. In consequence, meeting all criteria for creating an FI-CSHA with sufficient non-missing data meant that only 1,013 of 1,375 people with laboratory test information could be used. Even so, relaxing the criterion to allow up to 18 (of 38) variables to be missing (compared with the usual requirement for not calculating an FI for anyone for whom more than 20% of the items are missing) added another 158 subjects, without changing the properties of the resulting FI. Similarly, the mean age of the 362 people for whom we did not calculate an FI-LAB was 81.9 years (64.4% female) compared to 81.1 years (61.6% female) for those for whom we did calculate an FI-LAB. Likewise, the mean FI-CSHA score was 0.26 when all 1,375 subjects were used compared to 0.25 when only the 1,013 people investigated in this paper were considered. Still, understanding whether laboratory data will add value in more representative samples, or in other clinical samples, requires cross-validation in other datasets. In other settings, different or additional laboratory tests might be used reflecting local differences in relation to congenital and acquired disease, or those associated with lifestyle or environment (for example, thalassemia, histoplasmosis, alcoholism, air pollution). More recent reports might also change which tests were selected, for example, substituting alanine transaminase for aspartate transaminase [23–25].

Here, we tested the predictive validity of the frailty measures in relation to mortality. With each version of the FI, higher FI scores were associated with greater mortality, verified in a multivariable model that included age. Although death has the advantage of being a dichotomous, unambiguous and relevant example of an adverse outcome, not everyone dies a frail death. In consequence, it is important to distinguish between the exercise of predicting mortality *per se* and using it to validate the notion that more deficits are associated with greater risk. For example, were mortality prediction to be all that motivated our inquiry, then the FI would include chronological age, as evidenced by its persisting significance in each of the Cox proportional hazards models (Table 3). In the present context, this would, of course, be perverse: the goal of defining frailty is to address why, even though age is highly associated with the risk of adverse health outcomes (including death), not everyone of the same age has the same risk. The suggestion of the FI – that people with the most things wrong are at the highest risk – also has the advantage of being parsimonious, and of being sensible on its face, which itself is another form of validation [26].

The addition of laboratory test data also is of interest in understanding the associations between specific test abnormalities and frailty, as is commonly undertaken in inquiries about putative frailty mechanisms. For example, some groups have evaluated individual or even small numbers of laboratory tests [27–30]. A burgeoning literature on biomarkers, typically motivated by discrete mechanistic hypotheses, often considers such tests individually [31–34]. Our data suggest that any such results need to be interpreted in the context of the overall health state of the organism, if a general claim about frailty is to be made. Consider that the mean value of the FI score is typically closely related to age – or, as has been argued elsewhere – is a measure of biological age [10,35,36]. Aging is associated with a very large number of cellular and tissue mechanisms [37]. Finding associations with any single test abnormality can be helpful, but understanding where this fits in relation to other test abnormalities is an important step in aiming to gain a mechanistic understanding and in understanding systems effects. Our data suggest that, particularly for those sorts of inquiries, looking at the contribution of any single health deficit in isolation will be pragmatically difficult and theoretically dubious. A similar argument obtains in relation to understanding age-related mechanisms. Support for the latter comes from a recent study which shows that later life changes in myocyte structure and function are more closely tied to an FI than to chronological age [12]. As argued elsewhere, extending work on frailty to animal models can allow for exploration of mechanisms of both frailty and, more broadly, aging itself [38]. Finally, the observation here that more women had lower FI scores using the FI-LAB than using the FI-CSHA is of interest. Of note, either way women had lower mortality than did men for any level of either FI. The more conservative estimate of frailty status observed with the FI-LAB might be an explanation for the so-called male–female mortality-morbidity paradox [39]. This intriguing observation needs to be pursued further.

## Conclusions

The results of this study demonstrate that an FI constructed from routinely collected laboratory and clinical data identifies older adults at increased risk of death. A large number of additional inquiries are suggested by these current findings. Beyond replication, and as a probe for understanding mechanisms, the feasibility and utility of adding a large number of items to an FI using commonly evaluated laboratory tests might importantly advance routine frailty assessment, especially when these test results are used in conjunction with other relevant items from electronic medical records. These considerations are motivating additional inquiries by our group. In particular, further evaluation in clinical settings of adding routinely collected laboratory data to an FI is warranted.

## Additional file

Additional file 1: Figure S1. Number of people in each group, at baseline and follow-up. **Figure S2.** Receiver operating characteristic curves for each Frailty Index. **Table S1.** Items used in the FI-CSHA.

## Abbreviations

AUC: area under the curve
CI: confidence interval
CSHA: Canadian Study of Health and Aging
FI: frailty index
FI-CSHA: standard frailty index
FI-LAB: laboratory frailty index
ROC: receiver operating characteristic
