# Supplementary material for: Standard laboratory tests to identify older adults at increased risk of death
Source: BMC Med. 2014 Oct 7;12:171. doi: 10.1186/s12916-014-0171-9 (PMC4190374; doi:10.1186/s12916-014-0171-9)
Supplement: Additional file 1: Figure S1. — Number of people in each group, at baseline and follow-up. Figure S2. Receiver operating characteristic curves for each Frailty Index. Table S1. Items used in the FI-CSHA. [file 12916_2014_171_MOESM1_ESM.docx]

**Supplemental Figure 1: Number of people in each group, at baseline and follow-up.**

**
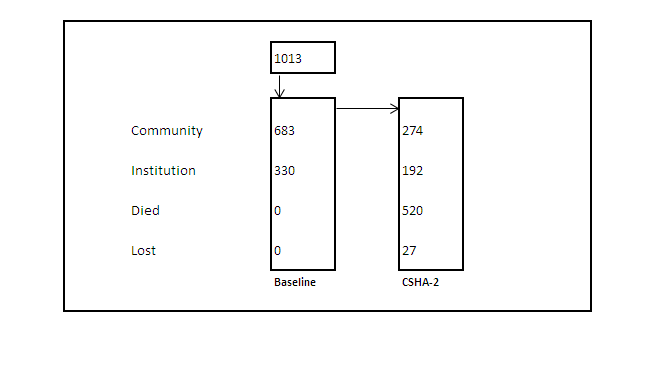
**

**Supplemental Figure 2: Receiver Operating Characteristic Curves for each Frailty Index**

**Supplemental Figure 2:** ROC (receiver operating characteristic) for FI-LAB (blue), AUC=0.72; FI-CSHA (green), AUC=0.73; Combined FI (red) AUC=0.74

**Supplemental Table 1: Items used in the FI-CSHA**

| **Frailty Index Variable** | **Health Deficit Scores** | | | | |
| --- | --- | --- | --- | --- | --- |
|  | **0** | **0.25** | **0.5** | **0.75** | **1** |
| 3MS | 79-100 |  | 49-78 |  | 0-48 |
| Self-Rated Health | Excellent | Very Good | Good | Fair | Poor |
| Anxiety | No |  |  |  | Yes |
| Depression | No |  |  |  | Yes |
| Other Psychiatric | No |  |  |  | Yes |
| Vision Problems | No |  |  |  | Yes |
| Hearing Problems | No |  |  |  | Yes |
| Speaking Problems | No |  |  |  | Yes |
| Walking Outside | Independent |  | Assisted |  | Dependent |
| Walking Indoors | Independent |  | Assisted |  | Dependent |
| Falls | No |  |  |  | Yes |
| Chair transfers | Independent |  | Assisted |  | Dependent |
| Weight Loss | No |  |  |  | Yes |
| Urinary Incontinence | No |  |  |  | Yes |
| Constipation | No |  |  |  | Yes |
| Shopping | Independent |  | Assisted |  | Dependent |
| Housework | Independent |  | Assisted |  | Dependent |
| Meals | Independent |  | Assisted |  | Dependent |
| Medications | Independent |  | Assisted |  | Dependent |
| Finances | Independent |  | Assisted |  | Dependent |
| Bathing | Independent |  | Assisted |  | Dependent |
| Dressing | Independent |  | Assisted |  | Dependent |
| Grooming | Independent |  | Assisted |  | Dependent |
| Toilet | Independent |  | Assisted |  | Dependent |
| Eating | Independent |  | Assisted |  | Dependent |
| High Blood Pressure | No |  |  |  | Yes |
| Heart Attack | No |  |  |  | Yes |
| CHF | No |  |  |  | Yes |
| Stroke | No |  |  |  | Yes |
| Cancer | No |  |  |  | Yes |
| Diabetes | No |  |  |  | Yes |
| Arthritis | No |  |  |  | Yes |
| Lungs | No |  |  |  | Yes |
| Kidneys | No |  |  |  | Yes |
| Sleeping Problems | No |  |  |  | Yes |
| Alcohol Problems | No |  |  |  | Yes |
| Other Medical | No |  |  |  | Yes |
| Medications | 0-4 |  | 5-9 |  | 10-14 |
